# Mutations in Known Monogenic High Bone Mass Loci Only Explain a Small Proportion of High Bone Mass Cases

**DOI:** 10.1002/jbmr.2706

**Published:** 2015-10-06

**Authors:** Celia L Gregson, Lawrie Wheeler, Sarah A Hardcastle, Louise H Appleton, Kathryn A Addison, Marieke Brugmans, Graeme R Clark, Kate A Ward, Margaret Paggiosi, Mike Stone, Joegi Thomas, Rohan Agarwal, Kenneth ES Poole, Eugene McCloskey, William D Fraser, Eleanor Williams, Alex N Bullock, George Davey Smith, Matthew A Brown, Jon H Tobias, Emma L Duncan

**Affiliations:** ^1^ Musculoskeletal Research Unit, School of Clinical SciencesUniversity of Bristol, Southmead HospitalBristolUK; ^2^ Human Genetics GroupUniversity of Queensland Diamantina Institute, Translational Research Institute, Princess Alexandra HospitalBrisbaneAustralia; ^3^ NIHR Oxford Musculoskeletal Biomedical Research UnitNuffield Orthopaedic CentreOxfordUK; ^4^ MRC Human Nutrition Research UnitElsie Widdowson LaboratoryCambridgeUK; ^5^ Mellanby Centre for Bone Research, Academic Unit of Bone MetabolismUniversity of SheffieldSheffieldUK; ^6^ Bone Research UnitUniversity Hospital Llandough, Cardiff and Vale University Health BoardCardiffUK; ^7^ James Paget University Hospital Foundation NHS TrustNorfolkUK; ^8^ Department of MedicineUniversity of CambridgeCambridgeUK; ^9^ Department of Medicine, Norwich Medical SchoolUniversity of East AngliaNorwichUK; ^10^ Structural Genomics ConsortiumUniversity of OxfordOxfordUK; ^11^ MRC Integrative Epidemiology UnitUniversity of BristolBristolUK; ^12^ Royal Brisbane and Women's HospitalBrisbaneAustralia

**Keywords:** LRP5, SOST, ANABOLIC, SEQUENCING, PROTEIN MODELING

## Abstract

High bone mass (HBM) can be an incidental clinical finding; however, monogenic HBM disorders (eg, *LRP5* or *SOST* mutations) are rare. We aimed to determine to what extent HBM is explained by mutations in known HBM genes. A total of 258 unrelated HBM cases were identified from a review of 335,115 DXA scans from 13 UK centers. Cases were assessed clinically and underwent sequencing of known anabolic HBM loci: *LRP5* (exons 2, 3, 4), *LRP4* (exons 25, 26), *SOST* (exons 1, 2, and the van Buchem's disease [VBD] 52‐kb intronic deletion 3′). Family members were assessed for HBM segregation with identified variants. Three‐dimensional protein models were constructed for identified variants. Two novel missense *LRP5* HBM mutations ([c.518C>T; p.Thr173Met], [c.796C>T; p.Arg266Cys]) were identified, plus three previously reported missense *LRP5* mutations ([c.593A>G; p.Asn198Ser], [c.724G>A; p.Ala242Thr], [c.266A>G; p.Gln89Arg]), associated with HBM in 11 adults from seven families. Individuals with *LRP5* HBM (∼prevalence 5/100,000) displayed a variable phenotype of skeletal dysplasia with increased trabecular BMD and cortical thickness on HRpQCT, and gynoid fat mass accumulation on DXA, compared with both non‐*LRP5* HBM and controls. One mostly asymptomatic woman carried a novel heterozygous nonsense *SOST* mutation (c.530C>A; p.Ser177X) predicted to prematurely truncate sclerostin. Protein modeling suggests the severity of the *LRP5*‐HBM phenotype corresponds to the degree of protein disruption and the consequent effect on SOST‐LRP5 binding. We predict p.Asn198Ser and p.Ala242Thr directly disrupt SOST binding; both correspond to severe HBM phenotypes (BMD *Z*‐scores +3.1 to +12.2, inability to float). Less disruptive structural alterations predicted from p.Arg266Cys, p.Thr173Met, and p.Gln89Arg were associated with less severe phenotypes (*Z*‐scores +2.4 to +6.2, ability to float). In conclusion, although mutations in known HBM loci may be asymptomatic, they only account for a very small proportion (∼3%) of HBM individuals, suggesting the great majority are explained by either unknown monogenic causes or polygenic inheritance. © 2015 The Authors *Journal of Bone and Mineral Research* published by Wiley Periodicals, Inc. on behalf of American Society for Bone and Mineral Research (ASBMR).

## Introduction

Worldwide, fewer than 30 families have been reported with low density lipoprotein receptor‐related protein 5 (*LRP5*) high bone mass (HBM) (MIM# 603506). LRP5, a ubiquitous cell membrane co‐receptor, mediates canonical Wnt signaling and, in bone, facilitates osteoblastic bone formation.^1^ The 10 documented gain‐of‐function *LRP5* mutations reported to date all lie in exons 2, 3, and 4, which collectively code for the first β‐propeller domain, reducing binding affinity with SOST (sclerostin) and Dickkopf 1.^2^, ^3^, ^4^, ^5^, ^6^, ^7^, ^8^, ^9^, ^10^, ^11^, ^12^, ^13^, ^14^, ^15^, ^16^, ^17^, ^18^ In contrast, loss‐of‐function *LRP5* mutations cause osteoporosis pseudoglioma syndrome (OPPG; MIM# 259770), an autosomal recessive condition of congenital blindness and severe childhood osteoporosis with skeletal fragility.^19^ Heterozygous carriers have been reported to have low bone mineral density (BMD).^20^ Most OPPG and low BMD–associated mutations have been described in the second and third β‐propeller domains.^16^ Loss‐of‐function *SOST* mutations cause sclerosteosis, a rare condition of excessive bone overgrowth (MIM# 269500); a downstream deletion is thought to be responsible for the milder phenotype of van Buchem's disease (VBD) (MIM# 239100).^21^, ^22^ Both *LRP5*‐related and *SOST*‐related HBM have been associated with bone overgrowth, mandible enlargement, oral tori, cortical thickening, poor buoyancy, and importantly, reduced fracture risk.^2^ Although sclerosteosis confers a severe phenotype with craniotomy occasionally required to relieve rising intracranial pressure from skull overgrowth, *LRP5* HBM has a variable phenotype and may be asymptomatic.^2^, ^3^, ^4^ Recently, *LRP4* mutations, thought to impair SOST‐LRP4 interaction, have been reported in a phenotype resembling sclerosteosis.^23^ Anti‐sclerostin antibodies are now in phase 3 clinical trials,^24^, ^25^ and other inhibitors of osteoblastic Wnt antagonists are in development as novel anabolic osteoporosis treatments.^26^, ^27^, ^28^, ^29^ Such developments exemplify the valuable insights gained from studying rare monogenic conditions. To date, however, no study has employed a systematic approach to establish the frequency or scope of HBM mutations and their associated phenotypes within the general population.

We have previously reported the clinical characteristics of a unique population of adults with unexplained HBM, identified from review of 335,115 historical dual‐energy X‐ray absorptiometry (DXA) scans across 13 UK National Health Service (NHS) centers for BMD *Z*‐scores or *T*‐scores ≥+4.^30^ Within this population, we aimed to determine the genetic causes of HBM by sequencing unrelated HBM cases for mutations in known anabolic HBM loci, namely *LRP5* (exons 2, 3, and 4), *LRP4* (exons 25 and 26), and *SOST* (exon 1, the coding region of exon 2, and the VBD 52‐kb intronic deletion occurring 35 kb downstream of *SOST*). We then aimed to determine the phenotypes associated with such mutations and relate these to predicted three‐dimensional protein models.

## Subjects and Methods

### Identification of HBM cases

The HBM study is a UK‐based multicentered observational study of adults with unexplained HBM, identified incidentally upon routine clinical DXA scanning. Full details of DXA database screening and participant recruitment have been reported^30^ (Supporting Information S1). Briefly, DXA databases containing 335,115 DXA scans were searched for individuals with a BMD *T*‐score or *Z*‐score ≥+4 at any site within the lumbar spine or hip, at 13 NHS hospitals in England and Wales (nine Hologic, four Lunar). A further two centers with Hologic scanners contributed 23 similar individuals identified prospectively. All 1505 DXA images with BMD *T*‐score or *Z*‐score ≥+4 were visually inspected; 962 cases with osteoarthritis and/or other causes of raised BMD were excluded (eg, surgical metalwork, Paget's disease, metastases^31^). A generalized HBM trait would be expected to affect both spine and hip BMD, though not necessarily to the same extent. Hence we refined our definition of HBM index cases as: (1) L_1_ *Z*‐score of ≥+3.2 plus total hip *Z*‐score of ≥+1.2; or (2) total hip *Z*‐score ≥+3.2 plus L_1_ *Z*‐score of ≥+1.2 (using age and gender‐adjusted BMD *Z*‐scores). A threshold of +3.2 was consistent with the only published precedent for identifying HBM using DXA;^3^ and also most appropriately differentiated generalized HBM from artifact.^32^ A standard deviation of +3.2 would be expected to identify a tail of 0.069% of a normal distribution.^33^

Of 533 unexplained HBM index cases invited, 258 (48.4%) agreed to participate.^30^ Index cases were asked to invite their first‐degree relatives and spouse/partner(s) to participate. HBM status was defined in first‐degree relatives as summed L_1_ plus total hip *Z*‐score of ≥+3.2.^30^ Family‐based controls comprised relatives with BMD below this threshold. HBM among spouses was defined as for index cases. Participants were excluded if under 18 years of age, pregnant, or unable to provide written informed consent for any reason. All participants were clinically assessed using a standardized structured history and examination, with phlebotomy for bone biochemistry, bone turnover markers (Supporting Information S2) and DNA collection. DXA scans were performed according to the manufacturer's standard scanning and positioning protocols. Where available, total body (TB) BMD, fat mass (FM) (including android and gynoid FM), and lean mass were measured as reported previously,^34^ and high‐resolution pQCT (HRpQCT) was performed (Supporting Information S2). Of note, no index cases who reported ever having fractured had radiologic, hematologic, or clinical features consistent with osteopetrosis.^30^ Written informed consent was obtained for all participants in line with the Declaration of Helsinki^35^ and this study was approved by the Bath Multi‐centre Research Ethics Committee (REC: 05/Q2001/78) and each NHS Local REC.

### Sanger sequencing for HBM mutations

DNA was extracted from peripheral venous blood using standard phenol/chloroform extraction. PCR amplification of *LRP5* (exons 2, 3, and 4), *LRP4* (exons 25 and 26), and *SOST* (exon 1, the coding region of exon 2, and the VBD‐associated 52‐kb intronic deletion 35 kb 3′ of *SOST*) was performed on 50 ng genomic DNA in a reaction mix consisting: 10× Immolase reaction buffer, 10 mM dNTPs, 50 mM MgCl_2_, 5 μM each primer, 0.5 U Immolase polymerase Taq (Bioline Reagents Ltd, London, UK), and water to final volume of 25 μL. PCR cycling conditions and primer sequences are shown in Supporting Information S3. Samples were Sanger sequenced using standard techniques (BigDye v3.1 chemistry; Life Technologies Corporation, Carlsbad, CA, USA), and capillary sequenced (3130 Genetic Analyzer; Life Technologies Corporation). Electrophoretograms were analyzed using sequence analyses software Genalys v2.0β.^36^ Variants were examined in online databases: NCBI dbGene/dbSNP (release 135), 1000 Genomes (www.1000genomes.org), UCSC (GRCh37/Hg19), Leiden Open Variation Database (LOVD) (www.lovd.nl), Exome Variant Server (http://evs.gs.washington.edu/EVS), and ExAC (http://exac.broadinstitute.org), using transcripts *LRP5*: ENSP00000294304, *SOST*: ENST00000301691, and *LRP4*: ENSG00000134569. We performed in silico functional prediction using PolyPhen,^37^ SIFT,^38^ PMut,^39^ and Mutation Taster^40^ bioinformatic prediction algorithms. When the same point mutation was identified in more than one family, haplotypes were compared between index case samples genotyped using an Infinium OmniExpress‐12v1.0 GWAS chip read using an Illumina iScan (Illumina, San Diego, CA, USA), with genotype clustering performed using Illumina BeadStudio software.

### Protein modeling

Structural models of the LRP5‐SOST complex were prepared from the homologous structure of the LRP6 complex (PDB code 3SOV).^41^ Mutations were introduced using the modeling program ICM‐Pro (Molsoft, San Diego, CA, USA) with local minimization to optimize side chain positions within 7 Å of the mutation site.^42^

### Statistical analysis

HBM cases were grouped by mutation type for quantitative analyses in comparison with (1) HBM cases lacking *LRP5/SOST/LRP4* mutations and (2) family controls. Descriptive statistics are presented as mean (95% confidence interval [CI]) for continuous and count (percentages) for categorical data. Linear regression was used to analyze continuous variables and generate standardized β coefficients and 95% CIs. Age, gender, menopausal status, and estrogen replacement therapy in women were considered a priori confounders; weight and height were additional potential confounders. Data were managed using Microsoft Access (Microsoft Corp., Redmond, WA, USA) (data entry checks; error rate <0.12%) and analyzed using Stata release 12 statistical software (StataCorp, College Station, TX, USA).

## Results

### LRP5

We identified two novel missense *LRP5* mutations ([c.518C>T; p.Thr173Met], [c.796C>T; p.Arg266Cys]) as well as three previously reported missense *LRP5* mutations ([c.593A>G; p.Asn198Ser], [c.724G>A; p.Ala242Thr], [c.266A>G; p.Gln89Arg]), associated with HBM in 11 adults among seven families (Table 1, Supporting Information S4). All *LRP5* mutations were heterozygous and segregated with HBM in available pedigrees (Supporting Information S5). Of 11 carrying a heterozygous *LRP5* mutation, none had sustained a low‐trauma or moderate‐trauma adult fracture, six reported an inability to float, seven had oral tori, and eight had a noticeably enlarged mandible (Table 1).

**Table 1:** Exonic Mutations Identified After Sanger Sequencing of all 258 HBM Index Cases With Clinical Characteristics and In Silico Functional Predictions

Pedigree	Gene	Mutation	Exon	Amino acid change	Age (years)	Gender	Z‐score total hip	Z‐score L_1_	Adult fracture	Enlarged mandible	Tori	Nerve compression	Sinks/ floats (S/F)	Polyphen	SIFT (score)	PMut	Mutation taster	GERP score
1	*LRP5*	593A>G	3	Asn198Ser	30	M	+12.2	+8.3	N	Y^a^	Y	N	S	Probably damaging	Damaging	Neutral	Disease‐causing	3.66
1	*LRP5*	593A>G	3	Asn198Ser	26	F	+6.8	+5.6	N	Y^a^	N	N	S	Probably damaging	Damaging	Neutral	Disease‐causing	3.66
1	*LRP5*	593A>G	3	Asn198Ser	66^b^	M	+10.2	+7.8	N	Y	Y	N	S	Probably damaging	Damaging	Neutral	Disease‐causing	3.66
2	*LRP5*	724G>A	4	Ala242Thr	49	F	+7.1	+10.7	N	Y	Y	N	S	Probably damaging	Damaging	Neutral	Disease‐causing	3.78
2	*LRP5*	724G>A	4	Ala242Thr	21	M	+6.4	+8.2	N	N	n/a	N	F	Probably damaging	Damaging	Neutral	Disease‐causing	3.78
3	*LRP5*	724G>A	4	Ala242Thr	21	F	+7.1	+6.0	N	Y^c^	Y	Y^d^	S	Probably damaging	Damaging	Neutral	Disease‐causing	3.78
4	*LRP5*	724G>A	4	Ala242Thr	64	F	+5.9	+8.1	N	Y	Y	Y^e^	S	Probably damaging	Damaging	Neutral	Disease‐causing	3.78
4	*LRP5*	724G>A	4	Ala242Thr	41	F	+3.1	+3.1	N	Y	Y	N	F	Probably damaging	Damaging	Neutral	Disease‐causing	3.78
5	*LRP5*	C796C>T^f^	4	Arg266Cys^f^	65	M	+2.5	+6.2	N	Y	Y	N	F	Probably damaging	Tolerated	Pathological	Disease‐causing	‐0.44
6	*LRP5*	266A>G	2	Gln89Arg	69^b^	M	+2.4	+4.6	N	N	N	Y^g^	n/a^h^	Benign	Tolerated	Neutral	Disease‐causing	4.48
7	*LRP5*	518C>T^f^	3	Thr173Met^f^	76	M	+3.6	+4.2	Y^i^	N	N	Y^j^	F	Probably damaging	Tolerated	Neutral	Disease‐causing	1.67
8	*SOST*	530C>A^f^	2	Ser177X^f^	70	F	+1.7	+3.5	N	Y	n/a	N	S	n/a	Tolerated	n/a	Disease‐causing	4.26

In silico functional predictions relate to decreases in antagonism of Wnt signaling and hence increased Wnt activity.

HBM = high bone mass; GERP = Genomic Evolutionary Rate Profiling; n/a = not available.

^a^ With prognatism.

^b^ History of glucocorticoid treatment with oral bisphosphonate use.

^c^ Enlarged and asymmetric.

^d^ Tightly packed brain gyri on MRI; cranial nerves V and VII mildly impaired.

^e^ Conductive deafness.

^f^ Novel HBM mutation.

^g^ Carpel tunnel syndrome.

^h^ Non‐swimmer.

^i^ Fibula aged 39, elbow aged 48, both very high impact fractures.

^j^ Ulna nerve decompression.

### LRP5 HBM quantitative analyses

The 11 HBM cases with *LRP5* mutations (“*LRP5* HBM cases”) were compared with 347 HBM cases without *LRP5* mutations (250 index cases, 94 affected first‐degree relatives, and three spouses who fulfilled HBM index case criteria) (“non‐*LRP5* HBM cases”), and 200 family controls. Eight and four HBM cases had TB DXA and HRpQCT performed respectively. *LRP5* HBM cases were taller than both non‐*LRP5* HBM cases and controls, with larger shoe size and substantially greater BMD at all measured sites, representing greater trabecular density and cortical thickness measured by HRpQCT (Table 2). *LRP5* HBM cases were also heavier than controls, with greater fat mass, particularly gynoid fat. After adjustment for age, gender, menopause, and estrogen replacement in women, the *LRP5* HBM cases remained substantially taller than both non‐*LRP5* HBM cases and controls (Supporting Information S6). Hence, analyses were further adjusted for height; *LRP5* HBM cases still had persistently greater BMD at all measured sites, as well as greater gynoid fat mass than controls (Table 3). Further adjustment for total weight highlighted a difference in gynoid fat mass between HBM cases and both controls and non‐*LRP5* HBM cases (Supporting Information S7). Although still within the normal reference range, after adjustment mean adjusted calcium, was higher among *LRP5* HBM cases; however, bone turnover marker levels were not discernibly different (Table 3).

**Table 2:** Clinical Characteristics of *LRP5* HBM Cases, Non‐*LRP5* HBM Cases, and Family Controls

	*LRP5* HBM (*n* = 11)	Non‐*LRP5* HBM (*n* = 347)	Controls (*n* = 200)
	Mean (SD)	Mean (SD)	Mean (SD)
Age (years)	49.5 (21.0)	61.9 (13.5)*	54.0 (16.1)
Height (cm)	178.1 (8.6)	166.3 (8.6)**	171.1 (10.2)**
Weight (kg)	93.7 (13.5)	84.8 (17.5)	82.4 (17.3)*
BMI (kg/m^2^)	29.5 (2.8)	30.7 (6.0)	28.0 (4.9)
Shoe size^a^	9.8 (2.3)	7.0 (1.9)**	7.9 (2.3)*
Total hip BMD Z‐score	5.8 (2.7)	3.0 (1.1)**	0.5 (0.9)**
L_1_ BMD Z‐score	6.4 (2.0)	3.9 (1.5)**	0.5 (1.2)**
TB BMD (mg/cm^2^)^b^	1.76 (0.32)	1.33 (0.10)**	1.21 (0.12)**
TB lean mass (kg)^b^	55.4 (11.7)	46.7 (10.1)*	51.3 (11.4)
TB fat mass (kg)^b^	37.9 (6.5)	35.4 (12.8)	29.0 (11.3)*
TB android fat mass (kg)^b^	3.72 (0.54)	3.43 (1.41)	2.90 (1.26)
TB gynoid Fat Mass (kg)^b^	6.64 (2.31)	5.64 (1.85)	4.85 (1.82)*
Glucose^c^	6.2 (3.8)	6.2 (2.2)	5.6 (1.3)
Adjusted calcium	2.45 (0.07)	2.41 (0.10)	2.40 (0.08)
Phosphate	1.12 (0.18)	1.37 (0.86)	1.17 (0.44)
Alkaline phosphatase	74.9 (17.7)	86.7 (33.6)	85.2 (33.9)
P1NP (µg/L)	44.1 (30.0)	36.3 (19.9)	41.1 (23.4)
CTX (µg/L)	0.23 (0.18)	0.19 (0.13)	0.24 (0.17)
Osteocalcin (total) (µg/L)	15.7 (8.0)	15.6 (7.8)	17.8 (7.8)
Tibial trabecular bone density (mg HA/cm^3^)^d^	313.1 (59.8)	215.1 (40.9)**	185 (41.5)**
Tibial number of trabeculae (1/mm)^d^	2.83 (0.24)	2.31 (0.28)*	2.25 (0.47)*
Tibial trabecular thickness (mm)^d^	0.09 (0.02)	0.08 (0.01)	0.07 (0.01)*
Tibial cortical thickness (mm)^d^	2.70 (1.07)	1.27 (0.43)**	1.18 (0.33)**
	n (%)	n (%)	n (%)
Female	5 (45.5)	269 (77.5)*	93 (65.8)
Postmenopausal	2 (40.0)	216 (80.3)*	48 (51.6)
Estrogen replacement use (ever)	1 (20.0)	127 (47.2)	15 (16.1)

HBM = high bone mass; TB = total body.

^a^ *n* = 468 for UK shoe size.

^b^ Total body DXA measures: *n* = 8 for *LRP5* HBM, 199 for non‐*LRP5* HBM, 126 for controls.

^c^ *n* = 247 for finger prick blood glucose.

^d^ HRpQCT measures: *n* = 4 for *LRP5* HBM, 59 for non‐*LRP5* HBM, 36 for controls. No *LRP5* HBM cases reported malignancy; 2 *LRP5* HBM cases, 7 non‐*LRP5* HBM cases, and 2 controls had ever used oral bisphosphonates.

*p < 0.05, **p < 0.001 when compared with *LRP5* HBM case.

**Table 3:** Clinical Characteristics of *LRP5* HBM Cases, Non‐*LRP5* HBM Cases, and Family Controls Adjusted for Age, Gender, Menopausal Status, and Estrogen Replacement Therapy in Women, and Height

	*LRP5* HBM (n = 11) mean (95% CI)	Non‐*LRP5* HBM (*n* = 347) mean (95% CI)	Controls (*n* = 200) mean (95% CI)
Shoe size^a^	8.04 (7.21–8.86)	7.47 (7.27–7.68)	7.25 (7.03–7.47)
Total hip BMD Z‐score	6.18 (5.43–6.94)	2.89 (2.71–3.08)**	0.54 (0.34–0.75)**
L_1_ BMD Z‐score	5.97 (5.08–6.87)	3.62 (3.4–3.84)**	0.42 (0.17–0.66)**
TB BMD (mg/cm^2^)^b^	1.70 (1.64–1.77)	1.35 (1.33–1.37)**	1.18 (1.16–1.20)**
TB lean mass (kg)^b^	48.2 (43.8–52.6)	49.4 (48.3–50.5)	47.5 (46.3–48.6)
TB fat mass (kg)^b^	36.4 (28.3–44.5)	35.3 (33.3–37.2)	30.3 (28.1–32.4)
TB android fat mass (kg)^b^	3.39 (2.44–4.33)	3.46 (3.22–3.69)	2.88 (2.62–3.13)
TB gynoid fat mass (kg)^b^	6.45 (5.29–7.61)	5.59 (5.31–5.87)	5.1 (4.79–5.41)*
Glucose^c^	6.1 (4.3–7.8)	6.2 (5.7–6.7)	6.0 (5.4–6.7)
Adjusted calcium	2.47 (2.42–2.53)	2.41 (2.39–2.42)*	2.41 (2.40–2.43)*
Phosphate	1.12 (0.72–1.51)	1.23 (1.14–1.33)	1.10 (0.99–1.20)
Alkaline phosphatase	74.1 (55.9–92.4)	81.0 (76.5–85.4)	84.2 (79.3–89.1)
P1NP (µg/L)	41.2 (28.5–54.0)	35.7 (32.6–38.8)	37.6 (34.2–41.1)
CTX (µg/L)	0.22 (0.12–0.31)	0.19 (0.17–0.22)	0.23 (0.20–0.25)
Osteocalcin (total) (µg/L)	18.4 (13.4–23.5)	17.1 (15.9–18.3)	19.5 (18.1–20.9)
Tibial trabecular bone density (mg HA/cm)^d^	296.4 (257.0–335.9)	210.9 (198.5–223.4)**	175.2 (161.8–188.7)**
Tibial number of trabeculae (1/mm)^d^	2.67 (2.33–3.00)	2.28 (2.17–2.38)*	2.17 (2.06–2.29)*
Tibial trabecular thickness (mm)^d^	0.09 (0.08–0.11)	0.08 (0.07–0.08)	0.07 (0.06–0.07)*
Tibial cortical thickness (mm)^d^	2.54 (2.18–2.91)	1.28 (1.16–1.39)**	1.05 (0.92–1.17)**

HBM = high bone mass; TB = total body.

^a^ *n* = 468 for UK shoe size.

^b^ Total body DXA measures: *n* = 8 for *LRP5* HBM, 199 for non‐*LRP5* HBM, 126 for controls.

^c^ *n* = 247 for finger‐prick blood glucose.

^d^ HRpQCT measures: *n* = 4 for *LRP5* HBM, 59 for non‐*LRP5* HBM, 36 for controls.

*p < 0.05, **p < 0.001 when compared with *LRP5* HBM cases.

### Individual LRP5 HBM Phenotypes

**Figure 1:**
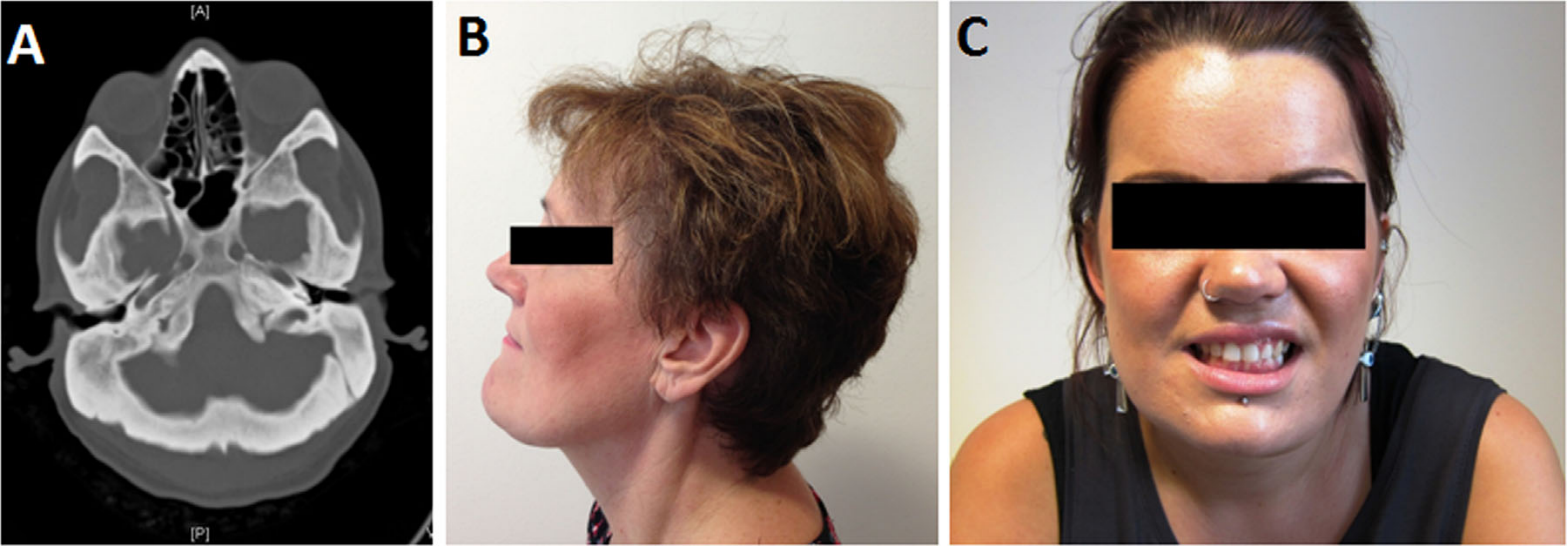
Clinical imaging in *LRP5* HBM. (*A*) Axial computed tomography image showing markedly thickened skull in male aged 30 years with p.Asn198Ser substitution (pedigree 1). (*B*) Mandible enlargement in female aged 49 years with p.Ala242Thr substitution (pedigree 2). (*C*) Asymmetric mandible enlargement with partial left cranial nerve V and VII impairment in female aged 21 years with p.Ala242Thr substitution (pedigree 3).

All clinical cases listed in Table 1 are described in detail in Supporting Information S7. Our most extreme HBM case, with femoral neck *T*‐score +12.2, had presented at age 19 years when he fainted and hit his head on a toilet seat, and in doing so broke the toilet seat; cranial imaging showed a markedly thickened skull (Fig. 1 *A*; Supporting Information S5). He has a heterozygous c.593A>G; p.Asn198Ser mutation that is predicted to be functionally deleterious by three of four in silico prediction tools; ie, suggesting decreased antagonism of Wnt signaling with subsequent increased Wnt activity (Table 1).

The most frequent *LRP5* mutation, c.724G>A; p.Ala242Thr, was identified in three unrelated families (with different *LRP5* haplotypes), explaining five HBM cases in total, and is predicted to be functionally deleterious by three of four *in silico* prediction tools (Table 1, Fig. 1 *B*, *C*). Interestingly, the novel mutation c.796C>T; p.Arg266Cys, was identified in a 65 year old man with a 25 year history of ulcerative colitis, for which he had been treated with glucocorticoids almost continuously for 21 years, despite which his HBM persisted; his bone turnover markers were normal (Supporting Information S5).

A heterozygous c.266A>G; p.Gln89Arg mutation was identified in an active man aged 69 years, with mild left hip osteoarthritis; he has never fractured. A novel c.518C>T; p.Thr173Met mutation was identified in a man aged 76 years, with osteoarthritis of knees, hands, and hips (unilateral hip replacement age 66 years), who had sustained two very‐high‐impact fractures age 39 years (fibula) and 48 years (elbow); the latter required ulna nerve decompression 18 years later with ongoing restrictions in the range of movement.

### LRP5 protein modeling

**Figure 2:**
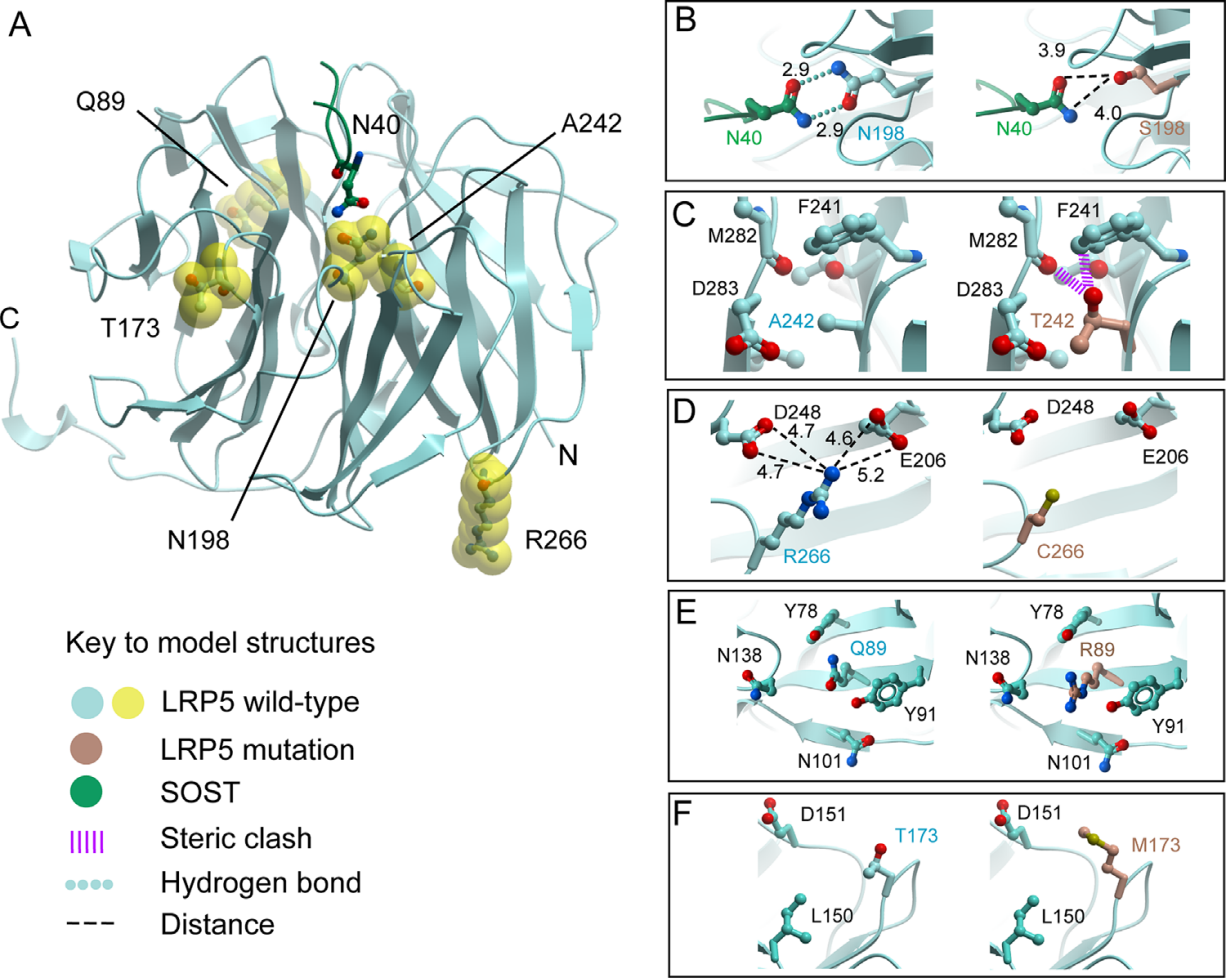
Structural models of the LRP5‐SOST complex (*A*) with mutations; p.Asn198Ser (*B*), p.Ala242Thr (*C*), p.Arg266Cys (*D*), p.Gln89Arg (*E*), and p.Thr173Met (*F*).

The p.Asn198Ser mutation directly affects the SOST interaction site and is predicted to disrupt SOST binding and inhibition, resulting in a severe HBM phenotype (Fig. 2 *A*). Modeling suggests that the shorter serine side chain is too distant to establish the two hydrogen bonds to SOST N40 (Asn40) that are formed by the wild‐type N198 (Asn198) side chain (Fig. 2 *B*). The similarly severe p.Ala242Thr mutation is predicted instead to disrupt the core packing of the LRP5 structure, thereby destabilizing the SOST binding site. The larger threonine side chain is likely to introduce steric clashes with the proximal F241 (Phe241) and M282 (Met282) (Fig. 2 *C*). The alanine at the LRP5 p.Ala242 position is conserved in LRP6 (p.Ala229), suggesting that tight packing in this region is favored. The site of the p.Ala242Thr mutation is only 10 Å from the SOST peptide binding site allowing even minor structural rearrangements to exert a negative effect on this interaction.

The mutations, p.Arg266Cys, p.Gln89Arg, and p.Thr173Met, were associated with less severe HBM phenotypes. p.Arg266 is located on the opposite face of the β‐propeller from the SOST binding site (Fig. 2 *A*) and is unlikely to directly disrupt its structure or to interfere directly with its binding (Fig. 2 *D*). Potentially, the introduction of an exposed cysteine residue could induce inappropriate disulphide bond formation resulting in misfolding and aberrant trafficking of the mutant protein.

The p.Gln89Arg mutation introduces an arginine side chain, which is found naturally at the equivalent position in wild‐type LRP6. However, the local packing around this site differs between the two proteins with the absence of a neighboring acidic residue in LRP5. The p.Gln89 position in LRP5 also appears more tightly packed due to the presence of both p.Y78 (Tyr78) and p.Y91 (Tyr91) (Fig. 2 *E*). Nonetheless, modeling suggests that the mutant arginine side chain can be tolerated. p.Gln89Arg is a reported SNP (rs41494349) with minor allele frequency (MAF) 0.02% in 1000 Genomes (0.1% MAF in East Asians, 0.005% in Europeans (ExAC)).

The p.Thr173Met mutation site lies in one of the β‐propeller loops that line the SOST binding site giving potential for disruption. However, p.Thr173 is distinct from the known peptide site and the side chain is likely to be oriented away from the peptide interface making no direct contact with it (Fig. 2 *F*). Moreover, the methionine substitution is predicted to be well tolerated. Thus, the less severe phenotypes associated with individuals carrying the p.Arg266Cys, p.Gln89Arg, and p.Thr173Met mutations may be due to less disruptive structural alterations. Models with DKK1 rather than SOST drew similar conclusions.

### SOST

We identified a novel heterozygous nonsense variant in exon 2 (c.530C>A; p.Ser177X) in a woman aged 70 years who reported difficulty floating (pedigree 8; Table 1). She had no symptoms of cranial nerve impingement, no syndactyly, and was 166 cm in height. This variant (cDNA.C577A, at chr17:41832822) is listed as rs143571358 in dbSNP135 and ExAC, with MAF of 0.04% and 0.0009% respectively. This base is highly conserved, with a genome evolutionary rate profiling score (GERP) of 4.26 (Table 1). rs143571358 has not been associated with any specific phenotype to date and remains unvalidated in dbSNP. This C>A variant lies within the coding sequence of *SOST*, and introduces a stop codon at p.177. This is predicted by Mutation Taster to be disease‐causing because the remaining 37 wild‐type amino acids are not incorporated in the mutated sclerostin protein, potentially causing nonsense‐mediated mRNA decay.

We also identified one previously reported SNP in exon 1 in eight HBM cases (rs17882143, c.28G>A; p.Val10Ile, MAF T = 0.047%), predicted to be benign by PolyPhen, tolerated by SIFT, a polymorphism by Mutation Taster, and neutral by PMut; as well as two intergenic SNPs in a further nine HBM cases (8 cases with rs28548107 MAF G = 0.046%; 1 case with rs181372199, MAF T = 0.043%). We found no variants in the 3′ regulatory region of *SOST* to suggest VBD. No *SOST* variants were identified in individuals with *LRP5* variants (and vice versa).

### LRP4

We found no mutations in *LRP4* exons 25 and 26. In one HBM case we identified a novel intronic heterozygous variant 24 bases before the start of exon 25 but not in the splice junction (c.3364‐24G>T). The common SNP rs2306033 (MAF 25% in 1000 Genomes) was observed in 56 (21%) HBM cases.

## Discussion

This study represents the largest systematic approach to date to identify the genetic cause of HBM, by screening all known HBM loci in a well‐defined population drawn from the general population, and has identified both novel and previously reported variants underlying HBM. We have increased to 13 the number of *LRP5* mutations associated with HBM, having identified two novel and three previously reported missense *LRP5* mutations, associated with HBM in seven families. The frequency of *LRP5* variants in HBM individuals, therefore, is 7 in 258, less than 3%; if our DXA population is representative of the general population, this extrapolates to an overall prevalence of *LRP5* HBM mutations in the UK of approximately 5 per 100,000. We identified one person with moderate HBM and a novel heterozygous nonsense *SOST* mutation predicted to prematurely truncate sclerostin, suggesting her to be a sclerosteosis carrier. However, no cases fulfilled a clear clinical diagnosis of autosomal recessive sclerosteosis with homozygous or compound heterozygous mutations. We did not observe any *LRP4* HBM variants in the limited number of exons sequenced. Our findings highlight the rarity of mutations in established HBM loci within the general population, and that the majority of HBM cases remain genetically unexplained.

The clinical variability we observed in *LRP5* HBM cases may arise from genotype/phenotype correlation, as suggested by the variable in silico functional consequences presented here. The *LRP5* p.Asn198Ser mutation, seen in our most extreme HBM case with hip BMD *Z*‐scores >+10, has been reported in a family with HBM and deafness, sensorimotor neuropathy, and spinal stenosis,^6^ features that we did not observe. Our protein modeling shows the direct implications of this mutation on SOST binding, explaining the extreme bone phenotype. A disrupted LRP5‐SOST binding site would be expected to lead to a relative resistance to sclerostin, with secondary increased sclerostin levels, an observation we have previously made in *LRP5* HBM.^43^ Our most frequent *LRP5* mutation (p.Ala242Thr), associated with BMD *Z*‐scores +3.1 to +10.7, has been reported in five previous families (two Portland US, one Sardinia, one France, one China),^7^, ^44^ to which we can now add a further three UK families.

We identified an isolated HBM case with an *LRP5* p.Arg266Cys variant; although having an allocated SNP ID, this SNP is not validated, has no described MAF in dbSNP, is not listed in ExAC/LOVD, and has not been described previously in association with HBM (or any phenotype). Three of four in silico prediction tools considered this variant to be functionally deleterious, although protein modeling suggested an indirect effect and/or altered protein folding. These less deleterious effects are consistent with a milder, mostly asymptomatic HBM phenotype (BMD *Z*‐scores +2.5 to +6.5).

*LRP5* mutation p.Gln89Arg was identified in one HBM case (no DNA was available from relatives). Interestingly p.Gln89Arg was also identified in one of the first reported *LRP5* HBM case series.^7^ However, this variant has been reported with MAF 8% in the Japanese population.^45^ Subsequently, p.Gln89Arg was associated with lower (not higher) femoral neck BMD in young Korean men (with MAF 19%)^46^ and postmenopausal Han Chinese women (with MAF 17%).^47^ However, this association with low BMD was not seen at the lumbar spine,^46^, ^47^ nor in 321 postmenopausal Japanese women, in whom it was instead associated with spinal osteophytes.^48^ Importantly functional analyses suggest that p.Gln89Arg does not play a functional role in canonical Wnt signaling.^49^ Whether p.Gln89Arg shares a rare haplotype with a functional BMD allele specifically in white populations is unknown.

*LRP5* mutation p.Thr173Met, identified in one isolated HBM case, was previously reported in association with abnormal retinal vasculature/folds in an older British woman diagnosed with familial exudative vitreoretinopathy (FEVR); however, her BMD was not reported.^50^ Although SIFT and PMut consider this mutation to be tolerated/neutral, respectively, PolyPhen and Mutation Taster predict it to be “probably damaging” and “disease causing,” with a moderate conservation score (GERP 1.67, Table 1). *LRP5* HBM mutations are considered fully penetrant; however, phenotypes may vary even within an individual family, as is seen in many genetic conditions; eg, osteogenesis imperfecta.

Originally LRP5 expression studies identified reduced affinity of DKK1 for LRP5 as the mechanism underlying *LRP5* HBM.^51^ The only previous LRP5 protein modeling to date (using p.Asn198Ser and p.Ala242Thr mutations, although without modeling the mutant side chains) predicted that both Wnt inhibitors DKK1 and SOST act through a common site in the first β‐propeller. This supports our models describing both LRP5‐SOST and LRP5‐DKK1 interactions.^41^ Furthermore, functional studies support p.Asn198Ser diminishing LRP5‐SOST binding; although LRP5‐DKK1 may be differentially affected.^52^ However, more recently SOST has emerged as the key LRP5 regulator.^53^ Genetic variance within both *SOST* and *LRP5* are associated with BMD in the general population, hence mechanistic insights can have application to the wider population.^54^, ^55^, ^56^ The extent to which common genetic variation in all BMD‐associated genes, explains the HBM phenotype in non‐*LRP5* HBM remains a question of interest.

Although absolute numbers are small, this remains one of the largest mutationally heterogeneous collections of *LRP5* HBM cases to date. Though the severity of the clinical phenotype varied according to the precise mutation, as a group, individuals with *LRP5* mutations appear to have a more extreme HBM phenotype when compared to other HBM individuals. We have previously reported that HBM individuals have greater trabecular BMD and cortical thickness as assessed by pQCT.^57^ In the present study we performed HRpQCT in a small subgroup; trabecular BMD and cortical thickness appeared to be higher in *LRP5* HBM compared to non‐LRP5 HBM cases. The increased sclerostin levels reported in *LRP5* HBM relative to other HBM cases, likely represents a compensatory response.^43^ Bone turnover appeared similar among *LRP5* HBM, non‐*LRP5* HBM, and controls (with low coefficients of variation).^34^ This null finding might reflect the small sample size. However, it is also likely that single time point sampling may not reflect accumulated life‐course exposure to anabolic stimuli. We lacked bone turnover assessment during peak bone mass accrual, during which time the effect of anabolic mutations might be expected to be maximal. Interestingly, two *LRP5* HBM cases (p.Arg266Cys; p.Asn198Ser) still had high BMD despite long‐term glucocorticoid use; whether anabolic *LRP5* mutations confer a relative resistance to glucocorticoid‐induced osteoporosis remains to be determined, but if so, it would be of great pharmacotherapeutic interest.

Fat mass was increased in individuals with *LRP5* HBM mutations, as we previously reported for HBM individuals overall.^34^ However, we observed a particular preponderance toward a gynoid fat mass distribution, persisting even after adjustment for body weight, in *LRP5* HBM compared with both non‐*LRP5* HBM and controls. This contrasts with the android fat phenotype we have previously indentified in HBM women (98% of whom have non‐*LRP5* HBM).^34^ Recent mechanistic analyses have shown *LRP5* HBM mutations, in some of the individuals reported here, lead to gluteofemoral fat accumulation due to altered LRP5‐dependent transcription in site‐specific depots, which raises interesting metabolic implications for medicines that modulate Wnt activity.^58^

Our study has limitations. The rarity of *LRP5* HBM challenged our sample size, restricted our ability to stratify (eg, by gender), and limited the confidence with which we can make prevalence estimates, it further prevented a clustered analysis, although evidence of intrafamilial clustering has previously been small.^30^ Furthermore, not all pedigrees had access to total body DXA and HRpQCT assessment, nor were full pedigrees able to be recruited in all cases. Interestingly, three spouses fulfilled HBM index case criteria, suggestive of assortative mating, as reported.^30^ Sequencing was restricted to exons in which anabolic mutations have been reported; and we have reported all identified variants. It is possible that variants in other β‐propeller regions may contribute to HBM; however, this seems less likely because mutations in the remaining β‐propellers have only been associated with FEVR and OPPG to date. Our approach was a practical one given the size of *LRP5* and exon 2 of *SOST*. We did not sequence for *DKK1* mutations; a novel missense mutation in the LRP5 inhibitor *DKK1* (c.74Y>F) has recently been reported to segregate with HBM in one Spanish family.^59^ Nor did we sequence *CLCN7* (associated with autosomal dominant osteopetrosis type 2) or genes associated with the more severe autosomal recessive forms of osteopetrosis (eg, *TNFSF11s*, *TCIRG1*, and *PLEKHM1*) because clinical and radiological phenotyping excluded diagnoses of osteopetrosis. The increasing availability and affordability of whole‐exome sequencing will allow comprehensive screening of all known anabolic and osteopetrotic loci simultaneously in similar future studies.

## Conclusions

We identified five missense *LRP5* mutations and one novel nonsense *SOST* mutation, in the largest population study of HBM to date. Protein modeling suggests the severity of high BMD corresponds to the degree of predicted LRP5 protein disruption. However, these *LRP5* and *SOST* HBM cases account for only a small proportion (∼3%) of HBM, raising the possibility that either mutations in novel HBM genes or polygenic inheritance is largely responsible for most cases of HBM in the population.

## Disclosures

All authors state that they have no conflicts of interest.

## Supporting information

Supporting Information.
