# Supplementary material for: Mutations in Known Monogenic High Bone Mass Loci Only Explain a Small Proportion of High Bone Mass Cases
Source: J Bone Miner Res. 2015 Oct 6;31(3):640–9. doi: 10.1002/jbmr.2706 (PMC4832273; doi:10.1002/jbmr.2706)
Supplement: Supplementary file 1 — Supporting Information. [file JBMR-31-640-s001.docx]

**Supporting Data**

**Contents**

**S1:** Flow diagram of recruitment

**S2:** Additional methods information (DXA, HRpQCT, Bone turnover markers)

**S3:** Primers for PCR amplification

**S4:** Electrophoretograms

**S5:** Clinical information and pedigrees for *LRP5* and non-*LRP5* HBM cases

**S6:** Clinical characteristics of *LRP5* High Bone Mass cases, non-*LRP5* HBM cases and family controls adjusted for age, gender, menopausal status, and estrogen replacement therapy in women

**S7:** Clinical characteristics of *LRP5* High Bone Mass cases, non-*LRP5* HBM cases and family controls adjusted for age, gender, menopausal status, and estrogen replacement therapy in women, height and weight

**Supporting Data S1**

**Flow diagram illustrating study recruitment**

**
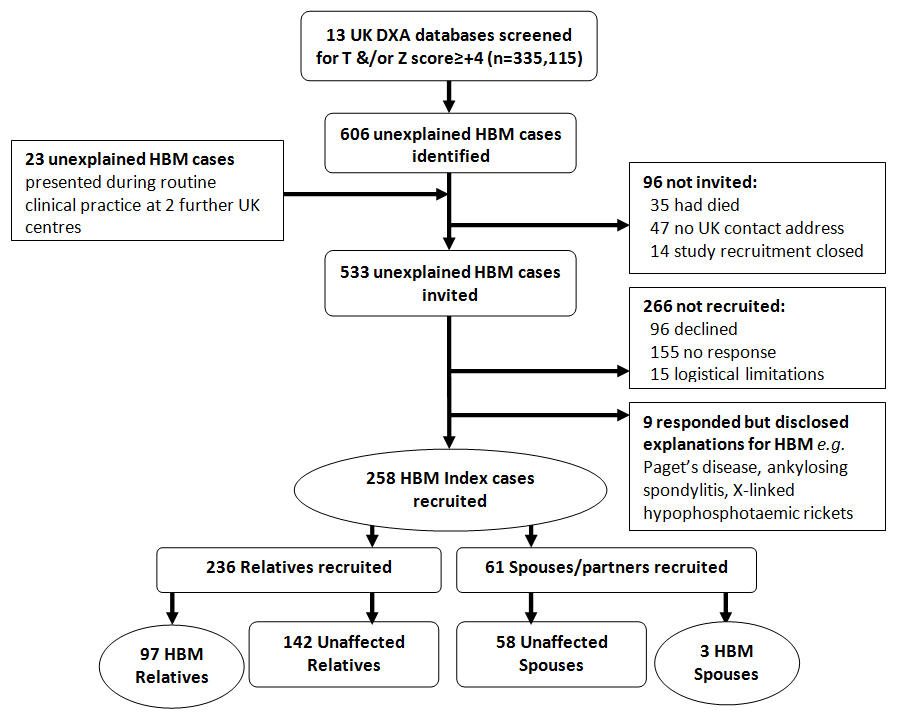
**

Three spouses fulfilled HBM index case criteria, suggestive of assortative mating as previously reported (1).

**Supporting Data S2**

**DXA measurements**

DXA scans were performed using either GE Lunar Prodigy DXA (software v.13.2, GE Healthcare, Madison, WI, USA), or Hologic Discovery/W DXA (software version Apex 3.0, Hologic Inc. Bedford, MA, USA). All scans were acquired and analysed according to each manufacturer’s standard scanning and positioning protocols. Total body (TB) BMD, fat mass (FM) (including specific android and gynoid regions of interest) and lean mass (LM) were measured, together with L1 and total hip BMD. Known differences in calibration exist between Hologic and GE for all scan type (2, 3). Systematic differences were limited using cross-calibration equations for all bone and soft tissue regions of interest (3, 4). Full details have previously been reported, including quality control checks and grading of TB scans for metallic artefact (5). TB scans with metallic artefacts were graded; this grade was added to all bone parameter regression models. Participants were centred for scanning; incomplete capture of soft tissue edges can introduce systematic bias as total body tissue is lost as body size increases. 89 (27%) scans had evidence of incomplete capture. Hence weighed weight (concurrently measured using scales) and calculated DXA weight were compared. Correlation (r^2^=0.984) and agreement (Bland-Altman rank correlation co-efficient r=-0.044, Pitman's Test of difference in variance p=0.426(23)) demonstrated no evidence of systematic bias. Hence further adjustment of analyses was not judged necessary. Known differences in calibration exist between Hologic and GE for all scan types (24;25). For lumbar spine and hip scans, systematic bias was limited by converting all measures to standardized BMD (sBMD) (26;27). For TB, systematic differences were limited using cross-calibration equations for all bone and soft tissue ROIs (25;28).

**HRpQCT measurements**

HRpQCT measurements of the distal tibia were performed using an XtremeCT (Scanco Medical, Zurich, Switzerland). A scout view was initially obtained to define anatomical landmarks within the region of interest and position the reference line, on the notch of the endplate of the distal tibia, to indicate the position of the first measurement slice. HR-pQCT scans were acquired in the high-resolution mode. Each scan resulted in the acquisition of a total of 110 image slices (stack height = 9.02 mm) at an isotropic resolution of 82 lm. All examinations were carried out on the non-dominant limb except when a participant had sustained a prior fracture of the non-dominant tibia, in which case the contra-lateral limb was measured. A maximum of one repeat scan was performed in the event of patient movement.

**Bone turnover markers**

Two non-fasted EDTA samples were collected and plasma separated and frozen within 4 hours to -80ºC. Bone formation (Procollagen type 1 amino-terminal propeptide [PINP], total osteocalcin) and resorption (β-C-telopeptides of type I collagen [CTX]) markers were also measured. All had inter- and intra-assay coefficients of variation <6.0% across the assay working ranges. Electrochemiluminescence immunoassays (ECLIA) (Roche Diagnostics, Lewes, UK) were used to measure plasma concentrations of PINP, osteocalcin, and CTX (detection limits 4.0, 0.6, 0.01μg/L respectively).

**Supporting Data S3**

**Primers for PCR amplification**

| Gene | Exon | Forward Primer (5’-3’) | Reverse Primer (5’-3’) | Annealing Temp (°C) |
| --- | --- | --- | --- | --- |
| LRP5 | 2 | TGT AAA ACG ACG GCC AGT CAT CCC AGG GCT GTG TAT CT | CAG GAA ACA GCT ATG ACC ACT TGG GCT CAT GCA AAT TC | 55 |
|  | 3 | CAG ATC TGT GTT AGC TGC TTC | CAG GAC TGC GTG GGT ACC TAC | 55 |
|  | 4 | GGG TCA GCA GCA ATG ACT GTC | CCA GAG CAT GGG CTT CTG CAG G | Touch-down |
| LRP4 | 25 | CTG CCA AGA GCT ATC CTG CT | TGT ACT GTC ACC CTG CGA GA | 60 |
|  | 26 | GGT ATG TGG TAG CTG CTG GAA | GAG ATC CTA GGC AGG CCT TT | 56 |
| SOST | 5’ reg. region | TAG AGA AAG ACC TCG TTA TTG G | TAC TAC TGG GCC TGG GAT GTA | 58 |
|  | 1 | GAG GGA AAC ATG GGA CCA G | ACT GTT CCT CGA CCA GTG CT | 58 |
|  | 2 | CTT TCC ACC AGC TCT AGA GC | CGC AGA GGA CAG AAA TGT GG | 58 |

Primers were designed using the program Primer3 (http://biotools.umassmed.edu/bioapps/primer3_www.cgi ) and assessed for specificity using the program BLAST (http://blast.ncbi.nlm.nih.gov/Blast.cgi)

**Supporting Data S4: Electrophoretograms**

**Electrophoretograms of mutations in exons 2, 3 and 4 of *LRP5* seen in 11 cases of High Bone Mass**

|  |  | **MUTATION** | **WILDTYPE** |
| --- | --- | --- | --- |
| Pedigree 1 | *c.A>G593;*  *Male aged 30* | 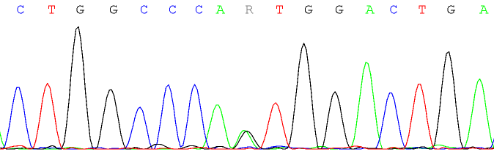 | 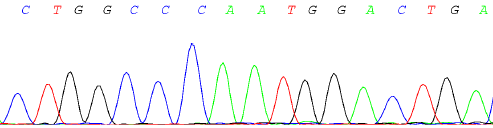 |
| Pedigree 1 | *c.A>G593;*  *Female aged 26* | 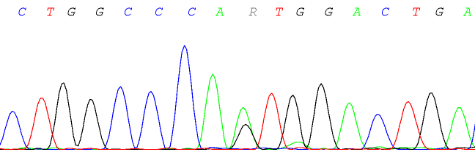 | 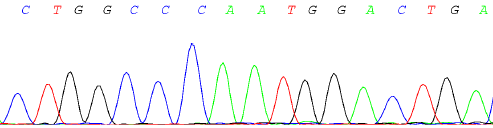 |
| Pedigree 1 | *c.A>G593;*  *Male aged 66* | 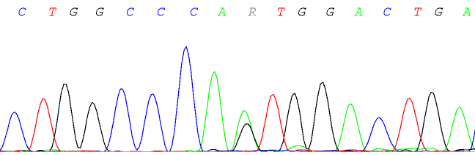 | 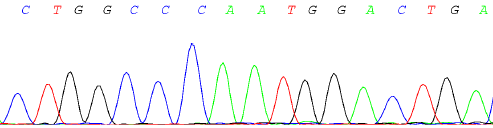 |
| Pedigree 2 | *c.G>A724;*  *Female aged 49* | 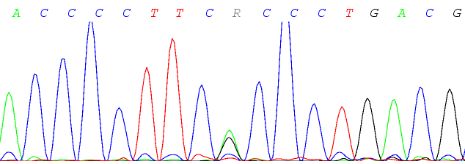 | 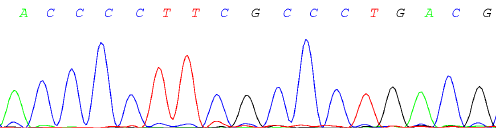 |
| Pedigree 2 | *c.G>A724;*  *Male aged 21* | 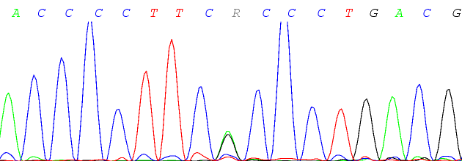 | 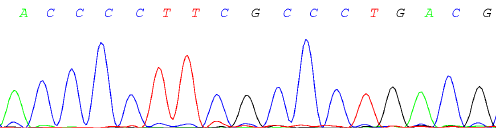 |
| Pedigree 3 | *c.G>A724;*  *Female aged 21* | 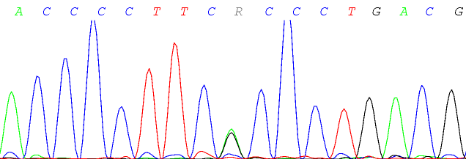 | 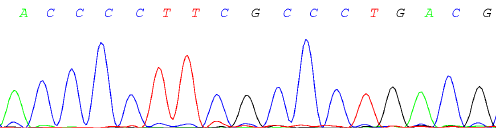 |
| Pedigree 4 | *c.G>A724;*  *Female aged 64* | 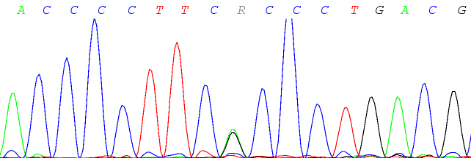 | 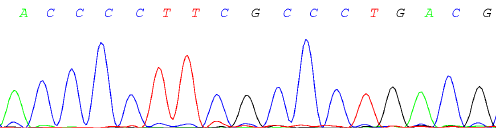 |
| Pedigree 4 | *c.G>A724;*  *Female aged 41* | 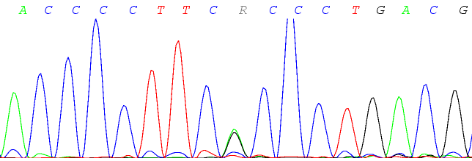 | 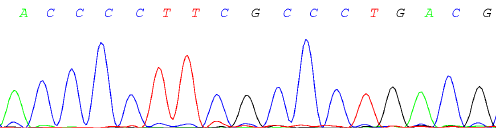 |
| Pedigree 5 | *c.C>T796;*  *Male age 65* | 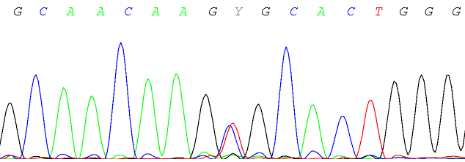 | 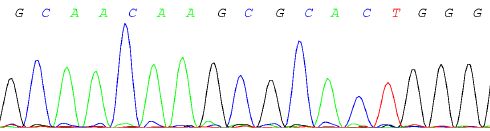 |
| Pedigree 6 | *c.A>G266;*  *Male aged 69* | 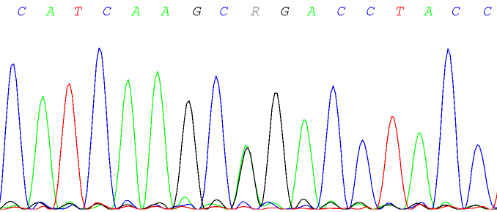 | 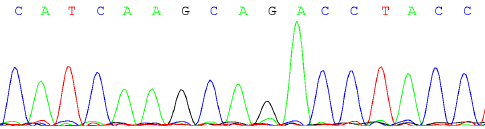 |
| Pedigree 7 | *c.C>T518;*  *Male aged 76* | 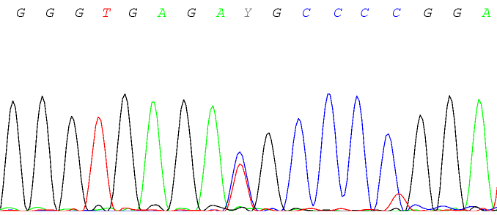 | 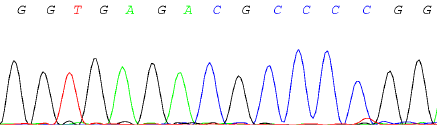 |

**Electrophoretogram of a mutation in exon 2 of *SOST* seen in 1 case of High Bone Mass**

|  |  | **MUTATION** | **WILDTYPE** |
| --- | --- | --- | --- |
| Pedigree 8 | *c.S>X177;*  *Female aged 70* | 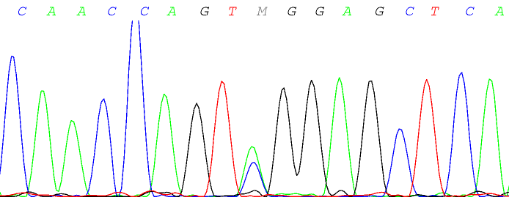 | 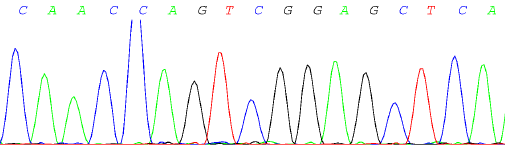 |

**Supporting Data S5: Clinical details regarding *LRP5* HBM cases**

Note: Legend for all pedigrees:

Circle symbol represents females, square symbol represents males. Filled symbols indicate individuals with HBM phenotype. Numbers inside symbols indicate the sum of L1 and total hip BMD Z-scores. Sequencing electrophoretograms are shown below affected individuals. WT = wildtype. ? = unknown phenotype and genotype.

**Pedigree 1: *LRP5* mutation c.593A>G; p.Asn198Ser**

The 30 year old male proband had first presented to hospital aged 19, having broken a toilet seat after fainting and hitting his head on it. A computerised tomography (CT) scan of the head excluded intracranial haemorrhage; the markedly thickened skull was overlooked (Figure 1A). Aged 24 he required osteotomy of an enlarged tibial tubercle and tendon release, having suffered with tibial tubercle tendonitis from age 19. He still suffers with mild shin pain. Aged 27 he was referred for chronic sternum pain related to a prominent sternum. Review of his historic CT head prompted referral to a metabolic bone clinic. He had an enlarged mandible with marked prognatism, bilateral 10mm torus mandibularis and mild dental overcrowding, despite orthodontic treatment as a child. He had an abnormal chest wall with prominence of left 3^rd^, 4^th^ and 5^th^ ribs with associated costochondral tenderness. He had a broad frame, varus knees, normal hearing (audiometry normal), normal joints and short sight requiring glasses. He is unable to float; rather he sinks rapidly, so avoids water.

His sister, four years younger, also reported difficulty floating and bilateral shin pain. She had recurrent ‘bony’ headaches, a wide deep mandible with prognatism but no tori, varus knees and minor asymptomatic lumbar scoliosis. She had had Graves’ disease at age 28, treated with radioiodine, and a history of depression controlled with fluoxetine.

Their 66 year old Afro-Caribbean father, who had never fractured, also reported difficulty floating. He too had a wide deep mandible with both torus mandibularis and palitinus. He had fixed flexion deformities in left and right elbows (10 and 20 degrees respectively) with asymptomatic reduced range of movement in both hips. He had type II diabetes mellitus, hypercholesterolaemia and hypertension. Interesting he had been diagnosed with myasthenia gravis three years earlier, and had been maintained on prednisolone 5-10mg since, with co-prescription of calcium and vitamin D supplementation and alendronic acid (which may explain his low bone turnover markers; P1NP 7.9 (reference: 20-76 ug/L); osteocalcin 5.0 (reference: 6.8-32.2 ug/L); serum CTX 0.02 (reference: 0.1-0.5 ug/L). His DXA scan identified substantially raised BMD. Their white European mother reported normal BMD.


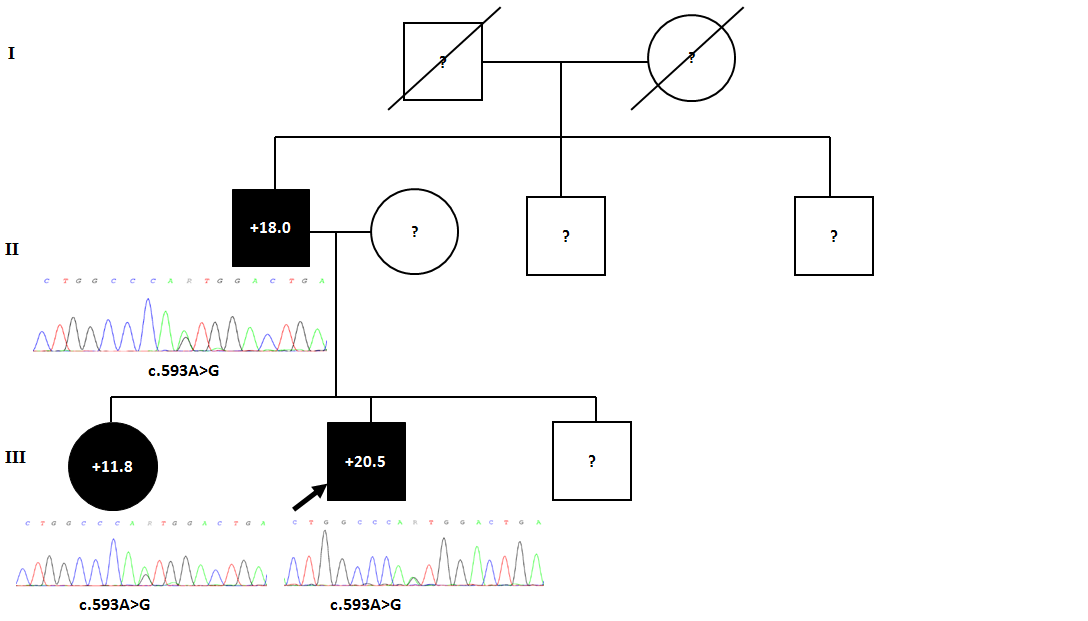
T-scores used in generation III as Z-scores not available

**Pedigree 2: *LRP5* mutation c.724G>A; p.Ala242Thr**

A 49 year old female proband reported being unable to float, and had felt self-conscious about her mandible enlargement (Figure 1B). She had torus palitinus and mandibularis with dental overcrowding. She had no symptoms or signs of joint disease, normal vision and audiometry, with a background of depression, including use of lithium for 15 years. One of her two sons also carried thec.724G>A mutation giving, at age 21, Z-scores Total hip +6.4, L1 +8.2. He had markers of increased bone turnover. Of note he has had nephrolithiasis with passage of renal calculi when aged 20 and 21.

**
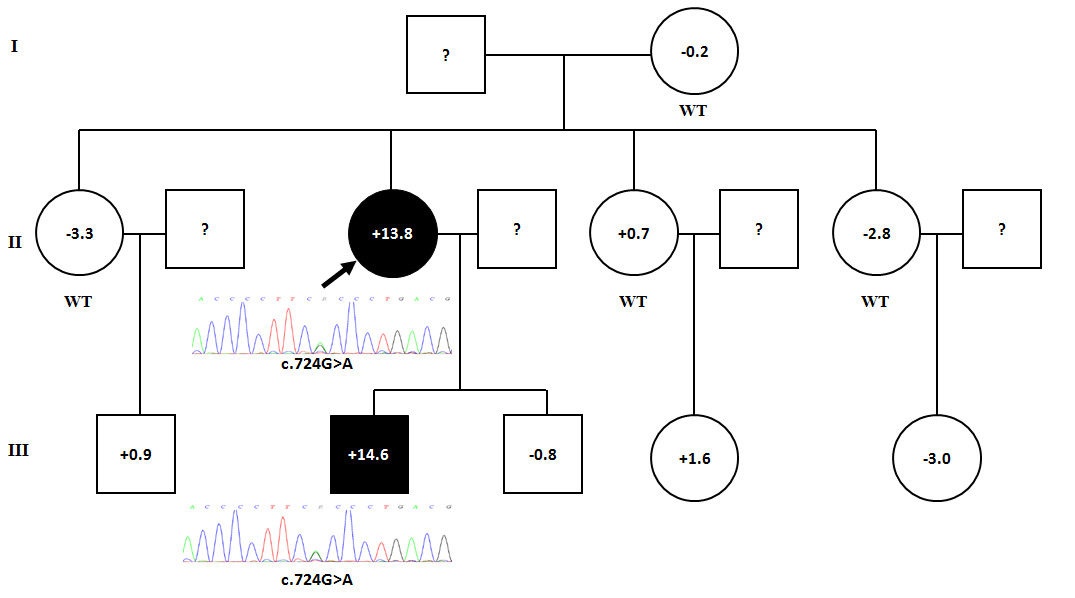
**

**Pedigree 3:** ***LRP5* mutation c.724G>A; p.Ala242Thr**

A 21 year old female had noticed her mandible enlarge with asymmetry developing over the previous 2 years, with associated bony pain (Figure 1C) for which she was under maxillo-facial review. She had both torus mandibularis and palitinus. She complained of bilateral shin pain, headaches for a year, pain in hips, lower back, wrists, left shoulder, for which she took paracetamol, tramadol, codeine, and naproxen and had previously been under rheumatology and pain team review. A magnetic resonance image (MRI) of her brain was reported as showing calvarium thickening resulting in tightly packed brain gyri and a ‘slightly misshapen’ brain. She had reduced sensation in the distribution of the left trigeminal cranial nerve, and mild left facial nerve weakness, but otherwise no neurological deficit (Figure 1C). Formal ophthalmological and auditory assessments were normal without evidence of nerve compression. Clinically she had no signs of osteoarthritis. Lumbar spine X-rays show hyperostosis in the supraspinous and anterio-longitudinal ligaments, the latter forming non-bridging anterior syndesmophytes. Lateral paravertebral osteophytes were present throughout the lumbar spine. Interestingly BMD testing in her parents and brother revealed normal BMD, suggesting this may a *de novo* mutation; unfortunately DNA was not available to test this hypothesis.


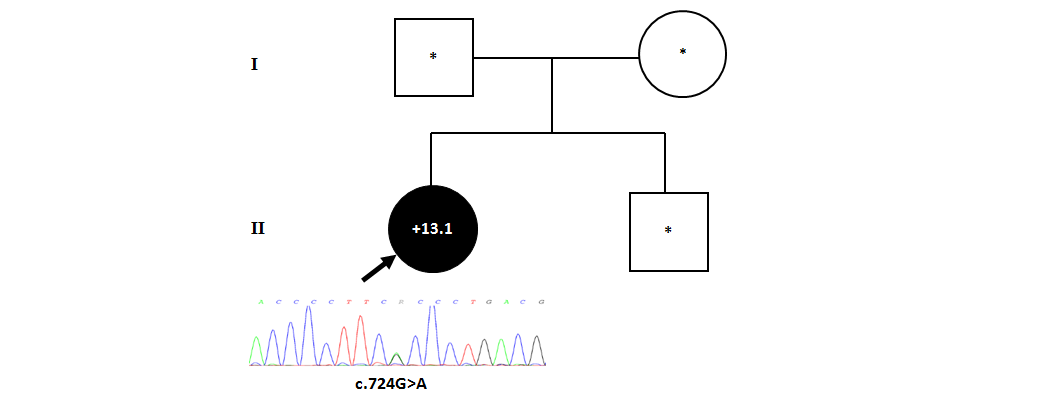


*Normal BMD, but Z-scores not disclosed

**Pedigree 4:** ***LRP5* mutation c.724G>A; p.Ala242Thr**

A 64 year old female reported a partial right-sided conductive deafness diagnosed 15 years earlier due to ‘build up of bone’. She was unable to float, had mandible enlargement with diffuse mandible pain, small bilateral torus mandibularis and dental overcrowding. She had a broad frame with symptoms and signs consistent with osteoarthritis of hips, knees, wrists, elbows, lumbar and cervical spine for which she takes paracetamol and gabapentin. She had previously had surgery to correct hallux valgus and hammer toes. Her history also included hypercholesterolemia, hypertension, and following the menopause, use of estrogen replacement for 10 years. One of her two daughters also carried the c.724G>A mutation with BMD at age 41 (and post-menopause) showingZ-scores total hip +3.1, L1 +3.1.


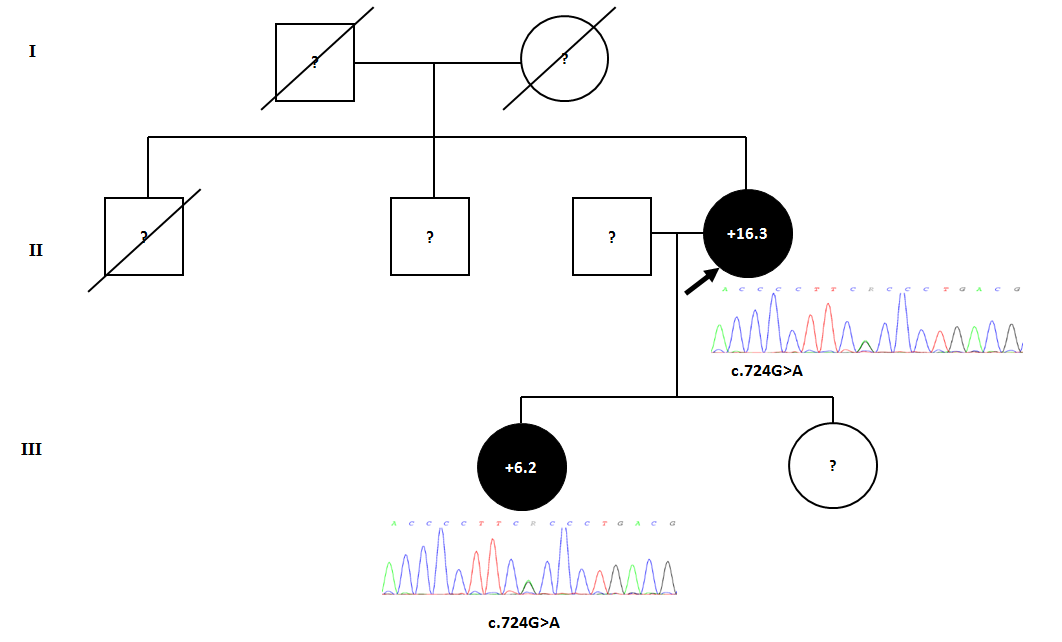


**Pedigree 5: *LRP5* mutation c.796C>T*;* p.Arg266Cys**

A novel mutation was identified in a 65 year old man with a 25 year history of ulcerative colitis for which he was awaiting colectomy. He had been treated with glucocorticoids almost continuously for 21 years, with a maximum dose at times of 40mg prednisolone/day; presently taking 10mg daily, together with risedronate (5mg daily). He had normal bone turnover; P1NP 21 (reference: 20-76 ug/L); osteocalcin 14.0 (reference: 6.8-32.2 ug/L); serum CTX 0.13 (reference: 0.1-0.5 ug/L). He had diet-controlled glucocorticoid-induced diabetes and clinical signs consistent with chronic steroid use. He had never fractured. He had a mildly increased mandible size, a broad frame, and both torus palitinus and mandibularis. No relatives were available to participate.

**Pedigree 6: *LRP5* mutation c.266A>G*;* p.Gln89Arg**

A mutation was identified in an active 69 year old man who had had a carpel tunnel release 5 years earlier (Table 2). He had mild right shoulder impingement and mild osteoarthritis of his left hip with asymptomatic bilateral knee crepitus. His hearing had been impaired since childhood, without further deterioration. He had type II diabetes mellitus hypercholestrolaemia, hypertension and gout. No relatives were available to participate.

**Pedigree 7: *LRP5 mutation c.518C>T;* p.Thr173Met**

This mutation was identified in a 76 year old man. He had sustained two very high impact fractures aged 39 (fibula) and 48 (elbow); the latter required ulna nerve decompression 18 years later though he still has ongoing restriction in range of movement. He had osteoarthritis of hips, knees and hands, with unilateral hip replacement aged 66, with a residual antalgic gait requiring use of a stick. He had a palpable enthesophyte at the right tibial tubercle and a left patellar enthesis. He had hypercholesterolemia, hypertension and angina. No relatives were available to participate.

**Supporting Data S6**: Clinical characteristics of *LRP5* High Bone Mass cases, non-*LRP5* HBM cases and family controls adjusted for age, gender, menopausal status, and estrogen replacement therapy in women

|  | ***LRP5* HBM (n=11)** | **Non-*LRP5* HBM (n=347)** | **Controls (n=200)** |
| --- | --- | --- | --- |
|  | **Mean (95% CI)** | **Mean (95% CI)** | **Mean (95% CI)** |
| **Shoe size ^a^** | 176.9 (172.4, 181.5) | 168.0 (166.9, 169.2)** | 167.5 (166.3, 168.7)** |
| **Total Hip Z-score** | 94.4 (83.1, 105.7) | 87.8 (85.0, 90.6) | 80.3 (77.2, 83.3)* |
| **L1 Z-score** | 30.1 (26.3, 33.9) | 31.1 (30.2, 32.1) | 28.6 (27.6, 29.6) |
| **TB BMD mg/cm^2 b^** | 9.2 (8.2, 10.2) | 7.4 (7.2, 7.7)* | 7.1 (6.9, 7.4)** |
| **TB Lean Mass (kg) ^b^** | 6.19 (5.45, 6.93) | 2.89 (2.71, 3.08)** | 0.54 (0.34, 0.75)** |
| **TB Fat Mass (kg)** ^b^ | 5.93 (5.06, 6.81) | 3.62 (3.40, 3.84)** | 0.42 (0.18, 0.66)** |
| **TB Android Fat Mass (kg)** ^b^ | 1.73 (1.66, 1.80) | 1.35 (1.33, 1.36)** | 1.18 (1.16, 1.20)** |
| **TB Gynoid Fat Mass (kg)** ^b^ | 52.5 (47.6, 57.4) | 49.3 (48.0, 50.5) | 47.2 (45.8, 48.5)* |
| **Glucose ^c^** | 38.2 (30.3, 46.2) | 35.2 (33.2, 37.2) | 30.1 (27.9, 32.3)* |
| **Corrected Calcium** | 89.4 (85.4, 93.5) | 91.8 (90.8, 92.8) | 91.9 (90.8, 93.0) |
| **Phosphate** | 275 (230, 321) | 264 (253, 275) | 288 (275, 300) |
| **Alkaline Phosphatase** | 8.5 (7.3, 9.8) | 7.0 (6.7, 7.3)* | 6.7 (6.4, 7.1)* |
| **P1NP (ug/L)** | 2.48 (2.43, 2.53) | 2.41 (2.39, 2.42)* | 2.41 (2.40, 2.43)* |
| **CTX (ug/L)** | 1.15 (0.76, 1.54) | 1.23 (1.14, 1.33) | 1.09 (0.99, 1.20) |
| **Osteocalcin (total) (ug/L)** | 71.4 (53.5, 89.3) | 81.0 (76.5, 85.4) | 84.4 (79.5, 89.3) |
| **Tibial Trabecular bone density (mg HA/cm) ^d^** | 296.8 (258.4, 335.1) | 210.9 (198.6, 223.2)** | 175.2 (161.9, 188.6)** |
| **Tibial No. of trabeculae (1/mm) ^d^** | 2.72 (2.4, 3.05) | 2.27 (2.16, 2.37)* | 2.18 (2.07, 2.29)* |
| **Tibial Trabecular thickness (mm) ^d^** | 0.09 (0.07, 0.10) | 0.08 (0.07, 0.08) | 0.07 (0.06, 0.07)* |
| **Tibial Cortical Thickness (mm) ^d^** | 2.51 (2.15, 2.86) | 1.28 (1.17, 1.40)** | 1.04 (0.92, 1.17)** |

HBM: High Bone Mass. SD: Standard Deviation. BMI: Body Mass Index. TB: Total Body. FM: Fat Mass, LM: Lean Mass. P1NP: N-terminal propeptides of type I procollagen, CTX: C- terminal cross-linking telopeptides of type I collagen. HA: hydroyapatite. ^a^n=468 for UK shoe size. ^b^Total body DXA measures n=8 for *LRP5* HBM, 199 for non-*LRP5* HBM, 126 for controls. ^c^n=247 for finger prick blood glucose. **^d^**HRpQCT measures n=4 for *LRP5* HBM, 59 for non-*LRP5* HBM, 36 for controls. *<0.05, **<0.001 when compared with LRP5 HBM cases

**Supporting Data S7**: Clinical characteristics of *LRP5* High Bone Mass cases, non-*LRP5* HBM cases and family controls adjusted for age, gender, menopausal status, and estrogen replacement therapy in women, height and weight

|  | ***LRP5* HBM (n=11)** | **Non-*LRP5* HBM (n=347)** | **Controls (n=200)** |
| --- | --- | --- | --- |
|  | **Mean (95% CI)** | **Mean (95% CI)** | **Mean (95% CI)** |
| **Shoe size ^a^** | 7.95 (7.17, 8.74) | 7.39 (7.20, 7.59) | 7.32 (7.11, 7.53) |
| **Total Hip Z-score** | 6.13 (5.39, 6.87) | 2.83 (2.65, 3.01)** | 0.59 (0.39, 0.80)** |
| **L1 Z-score** | 5.95 (5.06, 6.84) | 3.60 (3.38, 3.82)** | 0.44 (0.2, 0.69)** |
| **TB BMD mg/cm^2 b^** | 1.70 (1.63, 1.76) | 1.34 (1.32, 1.36)** | 1.19 (1.17, 1.20)** |
| **TB Lean Mass (kg) ^b^** | 47.1 (44.0, 50.2) | 48.4 (47.6, 49.1) | 48.4 (47.6, 49.2) |
| **TB Fat Mass (kg)** ^b^ | 33.9 (30.9, 36.9) | 32.7 (32.0, 33.5) | 32.5 (31.7, 33.3) |
| **TB Android Fat Mass (kg)** ^b^ | 3.11 (2.70, 3.52) | 3.16 (3.06, 3.26) | 3.13 (3.02, 3.24) |
| **TB Gynoid Fat Mass (kg)** ^b^ | 6.15 (5.46, 6.84) | 5.27 (5.10, 5.44)* | 5.38 (5.19, 5.57)* |
| **Glucose ^c^** | 6.06 (4.37, 7.75) | 6.09 (5.60, 6.59) | 6.18 (5.55, 6.80) |
| **Corrected Calcium** | 2.47 (2.42, 2.52) | 2.4 (2.39, 2.42)* | 2.42 (2.40, 2.43)* |
| **Phosphate** | 1.12 (0.72, 1.52) | 1.23 (1.14, 1.33) | 1.09 (0.99, 1.20) |
| **Alkaline Phosphatase** | 72.5 (55.0, 89.9) | 79.0 (74.7, 83.3) | 85.8 (81.1, 90.6) |
| **P1NP (ug/L)** | 41.1 (28.3, 53.9) | 35.6 (32.4, 38.7) | 37.7 (34.2, 41.2) |
| **CTX (ug/L)** | 0.22 (0.12, 0.31) | 0.19 (0.17, 0.22) | 0.23 (0.20, 0.25) |
| **Osteocalcin (total) (ug/L)** | 18.6 (13.5, 23.7) | 17.2 (16.0, 18.5) | 19.4 (18.0, 20.8) |
| **Tibial Trabecular bone density (mg HA/cm) ^d^** | 296.0 (257.1, 334.9) | 210.0 (197.7, 222.3)** | 177.3 (163.9, 190.8)** |
| **Tibial No. of trabeculae (1/mm) ^d^** | 2.66 (2.38, 2.93) | 2.25 (2.17, 2.34)* | 2.23 (2.13, 2.32)* |
| **Tibial Trabecular thickness (mm) ^d^** | 0.09 (0.08, 0.11) | 0.08 (0.07, 0.08) | 0.07 (0.06, 0.07)* |
| **Tibial Cortical Thickness (mm) ^d^** | 2.55 (2.18, 2.91) | 1.28 (1.17, 1.4)** | 1.04 (0.91, 1.16)** |

HBM: High Bone Mass. SD: Standard Deviation. BMI: Body Mass Index. TB: Total Body. FM: Fat Mass, LM: Lean Mass. P1NP: N-terminal propeptides of type I procollagen, CTX: C- terminal cross-linking telopeptides of type I collagen. HA: hydroyapatite. ^a^ n=468 for UK shoe size. ^b^ Total body DXA measures n=8 for *LRP5* HBM, 199 for non-*LRP5* HBM, 126 for controls. ^c^ n=247 for finger prick blood glucose. **^d^** HRpQCT measures n=4 for *LRP5* HBM, 59 for non-*LRP5* HBM, 36 for controls. *<0.05, **<0.001 when compared with LRP5 HBM cases

**References for Supporting Data**

1. Gregson CL, Steel SA, O'Rourke KP, Allan K, Ayuk J, Bhalla A, et al. 'Sink or swim': an evaluation of the clinical characteristics of individuals with high bone mass. Osteoporosis International. 2012;23(2):643-54.

2. Fan B, Lu Y, Genant H, Fuerst T, Shepherd J. Does standardized BMD still remove differences between Hologic and GE-Lunar state-of-the-art DXA systems? Osteoporosis International. 2010;21(7):1227-36.

3. Shepherd JA, Fan B, Wu XP, Ergun D, Levine M. Cross calibration of Whole Body Scans between GE-Lunar and Hologic Systems: Bone and Body composition. Journal of Clinical Densitometry. 2011;14(2):158.

4. Shepherd JA, Fan B, Lu Y, Wu XP, Wacker WK, Ergun DL, et al. A multinational study to develop universal standardization of whole body bone density and composition using GE Healthcare Lunar and Hologic DXA systems. Journal of Bone and Mineral Research. 2012;27(10):2208-16.

5. Gregson CL, Paggiosi MA, Crabtree N, Steel SA, McCloskey E, Duncan EL, et al. Analysis of body composition in individuals with high bone mass reveals a marked increase in fat mass in women but not men. J Clin Endocrinol Metab. 2013;98(2):818-28.
